# A bibliometric analysis of inflammatory bowel disease and COVID-19 researches

**DOI:** 10.3389/fpubh.2023.1039782

**Published:** 2023-01-30

**Authors:** Fangfei Wang, Jinliang Xie, Huifang Xiong, Yong Xie

**Affiliations:** ^1^Department of Gastroenterology, First Affiliated Hospital of Nanchang University, Nanchang, Jiangxi, China; ^2^Gastroenterology Institute of Jiangxi Province, Nanchang, Jiangxi, China; ^3^Key Laboratory of Digestive Diseases of Jiangxi Province, Nanchang, Jiangxi, China; ^4^Jiangxi Clinical Research Center for Gastroenterology, Nanchang, Jiangxi, China; ^5^Digestive Disease Hospital, First Affiliated Hospital of Nanchang University, Nanchang, Jiangxi, China

**Keywords:** bibliometric analysis, inflammatory bowel disease, COVID-19, trends, CiteSpace, VOSviewer

## Abstract

**Background:**

Patients with inflammatory bowel disease (IBD) often require immunosuppressive therapy and are hence susceptible to various opportunistic viral and bacterial infections. In this regard, many studies on IBD and COVID-19 have been conducted. However, no bibliometric analysis has been performed. This study provides a general overview of IBD and COVID-19.

**Methods:**

Publications about IBD and COVID-19 from 2020 to 2022 were retrieved from the Web of Science Core Collection (WoSCC) database. Bibliometric analysis was performed using VOSviewer, CiteSpace, and HistCite.

**Results:**

A total of 396 publications were retrieved and considered in this study. The maximum number of publications were from the United States, Italy, and England, and the contributions of these countries were significant. Kappelman ranked first in article citations. The Icahn School of Medicine at Mount Sinai and *Inflammatory Bowel Diseases* were the most prolific affiliation and journal, respectively. The most influential research topics were “management”, “impact”, “vaccination”, and “receptor”. The following keywords represented research frontiers: “depression”, “the quality of life of IBD patients”, “infliximab”, “COVID-19 vaccine”, and “second vaccination”.

**Conclusions:**

Over the past 3 years, most studies on IBD and COVID-19 have focused on clinical research. In particular, topics such as “depression”, “the quality of life of IBD patients”, “infliximab”, “COVID-19 vaccine”, and “second vaccination” were noted to have received much attention recently. Future research should focus on our understanding of the immune response to COVID-19 vaccination in biologically treated patients, the psychological impact of COVID-19, IBD management guidelines, and the long-term impact of COVID-19 in IBD patients. This study will provide researchers with a better understanding of research trends on IBD during COVID-19.

## 1. Introduction

COVID-19 was first detected in Wuhan, China, in late 2019. It evolved into a pandemic in the first quarter of 2020 after a substantial increase in the number of cases and the rapid geographic spread of the disease (1–3). COVID-19 is an infectious disease caused by the spread of severe acute respiratory syndrome coronavirus 2 (SARS-CoV-2) (4, 5). SARS-CoV-2 has a high mutation rate, and it has continued to evolve via mutation of its gene and protein sequences (6, 7). Several genetic variants of SARS-CoV-2, including Alpha, Delta, Omicron, and other variants, have altered the course of the COVID-19 pandemic (8–10). In addition, recombinant variants of SARS-CoV-2 are also being identified (11–15), which further causes an increase in transmissibility (12). On September 6, 2022, the WHO reported 603,164,436 confirmed COVID-19 cases and 6,482,338 deaths, with the numbers continuing to rise (16). Despite intensive research, a growing number of strains and mutation capabilities pose new challenges for governments and research communities. The pandemic continues to threaten public health worldwide.

Individuals with underlying diseases are more likely to have a severe course of COVID-19 infection (17, 18). In addition, COVID-19-related mortality is particularly high in older patients and those with chronic diseases (19). Inflammatory bowel disease (IBD) is a group of chronic diseases characterized by persistent inflammation of the gastrointestinal tract (20). Crohn's disease (CD) and ulcerative colitis (UC) are the most common forms of IBD. The incidence of IBD is increasing worldwide, with a high prevalence in developed countries (> 0.3% prevalence) (21). The cause of IBD is thought to be an impaired immune response, which is composed of an early defect in the innate immune response and a dysregulated T-cell response in the chronic stage of the disease (22). Therefore, patients with IBD are susceptible to COVID-19. Additionally, the therapy they receive significantly increases this risk. In clinical practice, three major classes of biologics are approved for IBD patients: tumor necrosis factor-alpha (TNF-α) antagonists, integrin, and interleukin-12/23 antagonists (23). The immune system of IBD patients may be compromised, and infections are likely to occur. Hence, studies on the relationship between IBD and COVID-19 are increasingly being investigated.

Recently, several scientific articles about the relationship between IBD and COVID-19 have been published. However, the development trend of such publications has not yet been systematically analyzed. Therefore, the publication trends of this area of research must be summarized urgently so that future studies can use this summary as a reference. The merging of scientific research and big data has created unprecedented opportunities for discovery, understanding, and innovation (24). Bibliometrics is a statistical method used to analyze research papers on a given topic mathematically (25). In this study, a comprehensive analysis of the literature on IBD and COVID-19 was conducted by searching the Web of Science Core Collection (WoSCC) database.

## 2. Materials and methods

### 2.1. Data sources

The WoSCC database is commonly used in bibliometric analysis (26–28). It provides comprehensive information and is regarded as the most influential database in bibliometrics.

### 2.2. Search strategies

The WoSCC database was searched on July 12, 2022, for all articles related to IBD and COVID-19, using the following search formula: TS = (“inflammatory bowel disease” OR “Ulcerative colitis” OR “Crohn's disease”) AND TS= (“Corona^*^” OR “2019-nCov” OR “nCov-19” OR “SARS-CoV2” OR “COVID^*^”). The retrieval time range was from January 1, 2020 to July 12, 2022.

### 2.3. Inclusion and exclusion criteria

The criteria for inclusion were as follows: (1) the manuscript focused on IBD and COVID-19, and all of its content was accessible; (2) the articles were included in the document types; and (3) the article was written in English. The exclusion criteria were as follows: (1) the main topics were not related to IBD or COVID-19; (2) abstracts, meeting news, briefings, etc., were published as articles. Based on the inclusion and exclusion criteria, two reviewers separately performed a full document review.

###  2.4. Bibliometric analysis

Two reviewers (FW and JX) independently sorted and selected the data from the obtained studies and then discussed what data should be included in the study. A total of 396 retrieved articles were automatically analyzed according to publication year, country/region, research institution, author, and journal. Microsoft Excel was used to track the statistics of the top countries/regions, research institutions, authors, and journals in terms of the number of publications, and the results were recorded in Excel documents. The H-index was used to evaluate the quality of the study. This index was introduced by Hirsch (29) to quantify the importance, significance, and impact of a scientist's cumulative research contributions (29). The total local citation score (TLCS) and the total global citation score (TGCS) were also calculated. The HistCite tool was used to analyze the TLCS and TCGS. The TLCS is the number of times a set of papers included in a collection has been cited by other papers within the collection; the TGCS is the number of times a set of papers has been cited in the WoSCC database (30).

### 2.5. Visualize analysis

VOSviewer software, produced by Leiden College in the Netherlands, was used to visually analyze the publication data, and collaboration analysis between institutes, countries, and regions, author citation analysis, and journal citation analysis were performed. CiteSpace by Chaomei Chen was used to perform reference co-citation, keyword co-occurrence, and keyword burst analyses. All downloaded txt files were imported into CiteSpace, and the timing was set to January 1, 2020–July 12, 2022. The Pathfinder algorithm was selected, and finally, a classic view was created.

## 3. Results

### 3.1. General data

The amount of attention that the literature related to IBD and COVID-19 received was determined based on the number of articles. As of July 12, 2022, an initial search identified 1,555 studies. Among them, “Article” had 795 entries. After excluding studies that did not meet the language criterion, 396 studies remained. The total number of citations was 3,899, and the average number per paper was 9.85. The H-index was 27. Supplementary Table S1 lists the 10 most frequently cited publications. “Corticosteroids, but not TNF antagonists, are associated with adverse COVID-19 outcomes in patients with inflammatory bowel diseases: results from an international registry (31)” by Ungaro RC et al. had the highest number of total citations.

### 3.2. Countries/regions and institutions

The total number of countries/regions that published papers on IBD and COVID-19 was 59, of which 18 published more than 10 papers. Table 1 lists the 15 countries/regions with the highest publication volume. Among them, the United States had the maximum number of publications, with 132 (33.333%) articles, followed by Italy and England with 67 (16.919%) and 59 (14.899%) articles, respectively. The United States had the highest TLCS and TGCS, followed by Italy and England in turn.

**Table 1:** The top 15 countries/regions in terms of publication volume.

**Rank**	**Country/Region**	**Count**	**Proportion (%)**	**H-index**	**TLCS/TGCS**
1	USA	132	33.333	19	92/1946
2	Italy	67	16.919	15	57/758
3	England	59	14.899	14	38/892
4	Peoples R China	35	8.838	10	11/934
5	Germany	31	7.828	10	21/513
6	Spain	30	7.576	10	14/380
7	Canada	27	6.818	9	19/932
8	Australia	21	5.303	6	2/106
9	France	21	5.303	10	13/708
10	Netherlands	20	5.051	5	3/152
11	Israel	17	4.293	6	2/190
12	Scotland	16	4.040	9	10/552
13	Portugal	14	3.535	8	4/220
14	Denmark	13	3.283	4	5/85
15	Japan	13	3.283	3	0/31

TLCS, Total local citation score, which is the number of times cited by other papers in the local collection; TGCS, Total global citation score, which is the citation frequency based on the full WoSCC count at the time the data was downloaded.

Table 2 lists the 10 most active institutions. The Icahn School of Medicine at Mount Sinai and the University of North Carolina published the maximum number of articles (21 articles). Harvard University and the University of North Carolina Chapel Hill were second (20 articles), followed by the University of London (19 articles) and the Imperial College London (16 articles). All institutions were from Western countries. The Icahn School of Medicine at Mount Sinai ranked the highest in the TLCS and TGCS, followed by the University of North Carolina and the University of North Carolina Chapel Hill. The Icahn School of Medicine at Mount Sinai is considered the leading institute in terms of paper and article quality.

**Table 2:** The top 10 activist institutions.

**Rank**	**Institution**	**Count**	**Country/Region**	**H-index**	**TLCS/TGCS**
1	Icahn School of Medicine at Mount Sinai	21	USA	8	23/849
2	University of North Carolina	21	USA	8	18/809
3	Harvard University	20	USA	10	5/375
4	University of North Carolina Chapel Hill	20	USA	8	18/809
5	University of London	19	England	11	10/556
6	Imperial College London	16	England	8	18/445
7	Harvard Medicine School	14	USA	6	0/146
8	Mayo Clinic	13	USA	5	3/321
9	University of Pennsylvania	13	USA	7	8/718
10	Barts Health NHS Trust	12	England	7	9/410

TLCS, Total local citation score, which is the number of times cited by other papers in the local collection; TGCS, Total global citation score, which is the citation frequency based on the full WoSCC count at the time the data was downloaded.

### 3.3. Authors and journals

Table 3 lists the 10 most prolific authors. Ungaro RC (17 publications) published the highest number of papers, followed by Kappelman MD (14 publications) and Colombel JF (13 publications). Zhang X ranked fourth, Brenner EJ ranked fifth, Danese S ranked sixth, and Reinisch W ranked seventh. In all, 6 authors were from the United States, 1 from Italy, 1 from Canada, 1 from England, and 1 from Denmark. Kappelman MD had the most connections, with 762 citations. Other highly cited authors were Ungaro RC, Colombel JF, Zhang X, and Brenner EJ. Therefore, these authors were pioneers of research on IBD and COVID-19 and played an important role in opening up new research areas and topics.

**Table 3:** The 10 most productive authors.

**Rank**	**Author**	**Count**	**Country/Region**	**Institution**	**H-index**	**TLCS/TGCS**
1	Ungaro RC	17	USA	Icahn School of Medicine at Mount Sinai	8	21/750
2	Kappelman MD	14	USA	University of North Carolina at Chapel Hill	8	15/762
3	Colombel JF	13	USA	Icahn School of Medicine at Mount Sinai	7	17/716
4	Zhang X	11	USA	University of North Carolina at Chapel Hill	6	10/674
5	Brenner EJ	10	USA	University of North Carolina at Chapel Hill	6	12/656
6	Danese S	10	Italy	IRCCS Ospedale San Raffaele and University Vita-Salute San Raffaele	4	2/100
7	Reinisch W	10	Canada	Medical University of Vienna	5	10/628
8	Sebastian S	10	England	Hull University Teaching Hospitals NHS Trust	7	13/416
9	Farraye FA	9	USA	Mayo Clinic	5	6/74
10	Agrawal M	8	Denmark	Aalborg University	5	0/82

TLCS, Total local citation score, which is the number of times cited by other papers in the local collection; TGCS, Total global citation score, which is the citation frequency based on the full WoSCC count at the time the data was downloaded.

Table 4 lists the 15 most popular journals by the number of articles published. *Inflammatory Bowel Diseases* published the most studies, with 38 publications. The *Journal of Crohn's and Colitis* published 36 articles, the *Journal of Clinical Medicine* published 15, *Digestive and Liver Disease* published 10, and the *American Journal of Gastroenterology* and *Frontiers in Medicine* published 9. *Gut* had the highest GCS, followed by the *Journal of Crohn's & Colitis, Gastroenterology*, and *Inflammatory Bowel Diseases*. Finally, the *Journal of Crohn's and Colitis* had the highest LCS, followed by *Inflammatory Bowel Diseases*.

**Table 4:** The 15 most popular journals by number of articles published.

**Rank**	**Journal**	**Count**	**Impact Factor (2021)**	**H-index**	**TLCS/TGCS**
1	*Inflammatory bowel diseases*	38	7.29	10	10/245
2	*Journal of crohns and colitis*	36	10.02	9	15/442
3	*Journal of clinical medicine*	15	4.964	4	0/54
4	*Digestive diseases and sciences*	10	3.487	3	0/25
5	*American journal of gastroenterology*	9	12.045	5	0/83
6	*Frontiers in medicine*	9	5.058	3	0/28
7	*Digestive and liver disease*	8	5.165	6	2/82
8	*Gut*	7	31.795	7	2/658
9	*Journal of gastroenterology and hepatology*	7	4.369	4	0/43
10	*European journal of gastroenterology hepatology*	6	2.586	2	0/12
11	*Frontiers in immunology*	6	8.787	2	0/40
12	*Gastroenterology*	6	33.883	7	0/419
13	*Vaccines*	6	4.961	2	0/16
14	*Alimentary pharmacology therapeutics*	5	9.524	3	1/134
15	*BMJ open*	5	3.007	2	0/10

TLCS, Total local citation score, which is the number of times cited by other papers in the local collection; TGCS, Total global citation score, which is the citation frequency based on the full WoSCC count at the time the data was downloaded.

### 3.4. Cooperative countries/regions, institutions and authors

**Figure 1:**
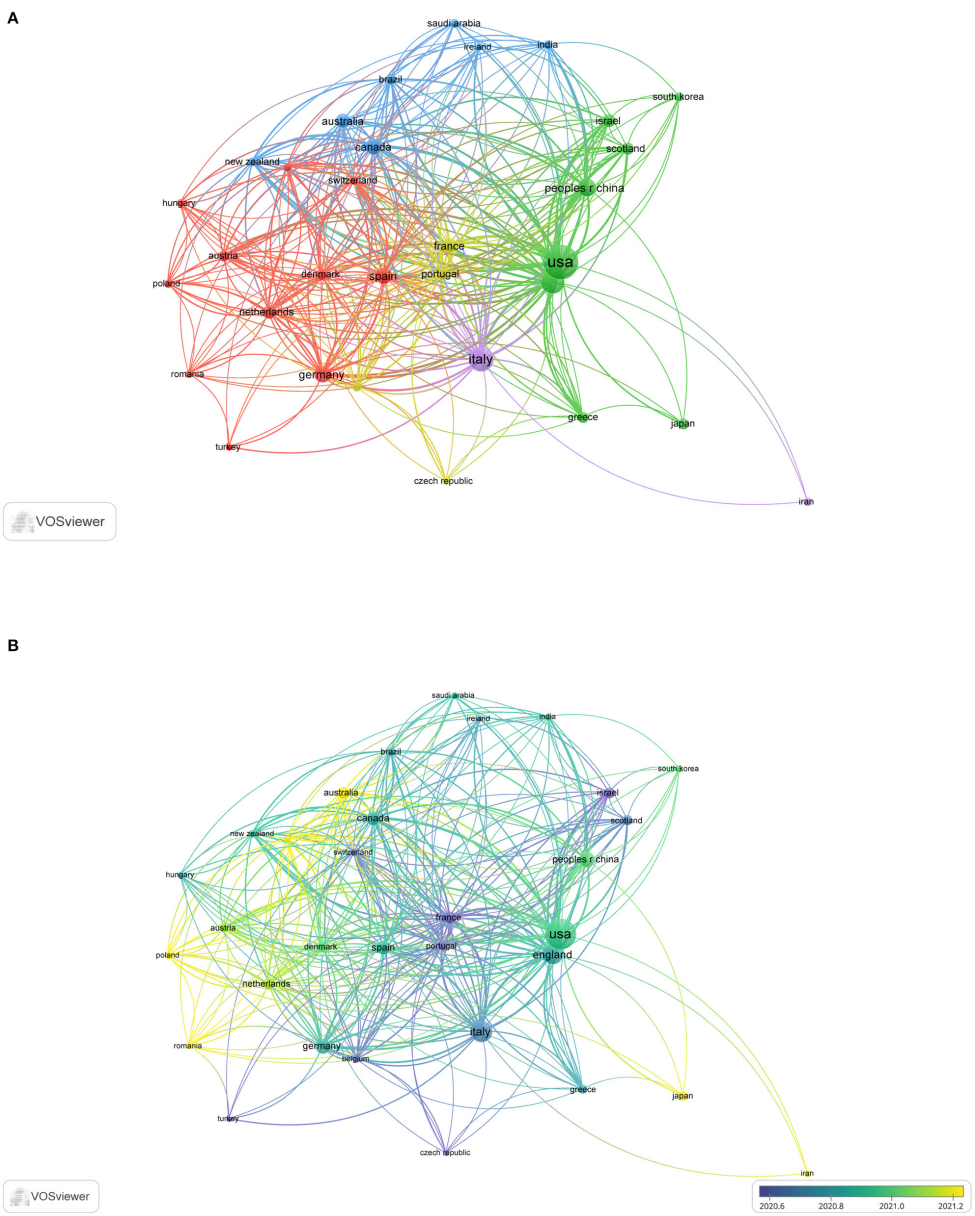
The cooperative countries in the field of IBD and COVID-19. **(A)** The top 32 most cooperative countries. **(B)** Time sequence chart of cooperative countries.

Figure 1A shows the top 32 most cooperative countries that have published documents on IBD and COVID-19. The minimum number of documents published by a country was 5. The cooperation network graph depicted in the figure is contiguous, with no isolated point. The nodes are divided into four clusters according to their colors. Countries in the same cluster cooperate closely. Western countries are at the center of the network, with more publications than Eastern countries. Figure 1B shows that France, Portugal, Italy, Belgium, Switzerland, Israel, Scotland, and other Western countries established cooperative relations earlier. Australia established close cooperation with Poland, Romania, Japan, and Iran approximately 2021.

**Figure 2:**
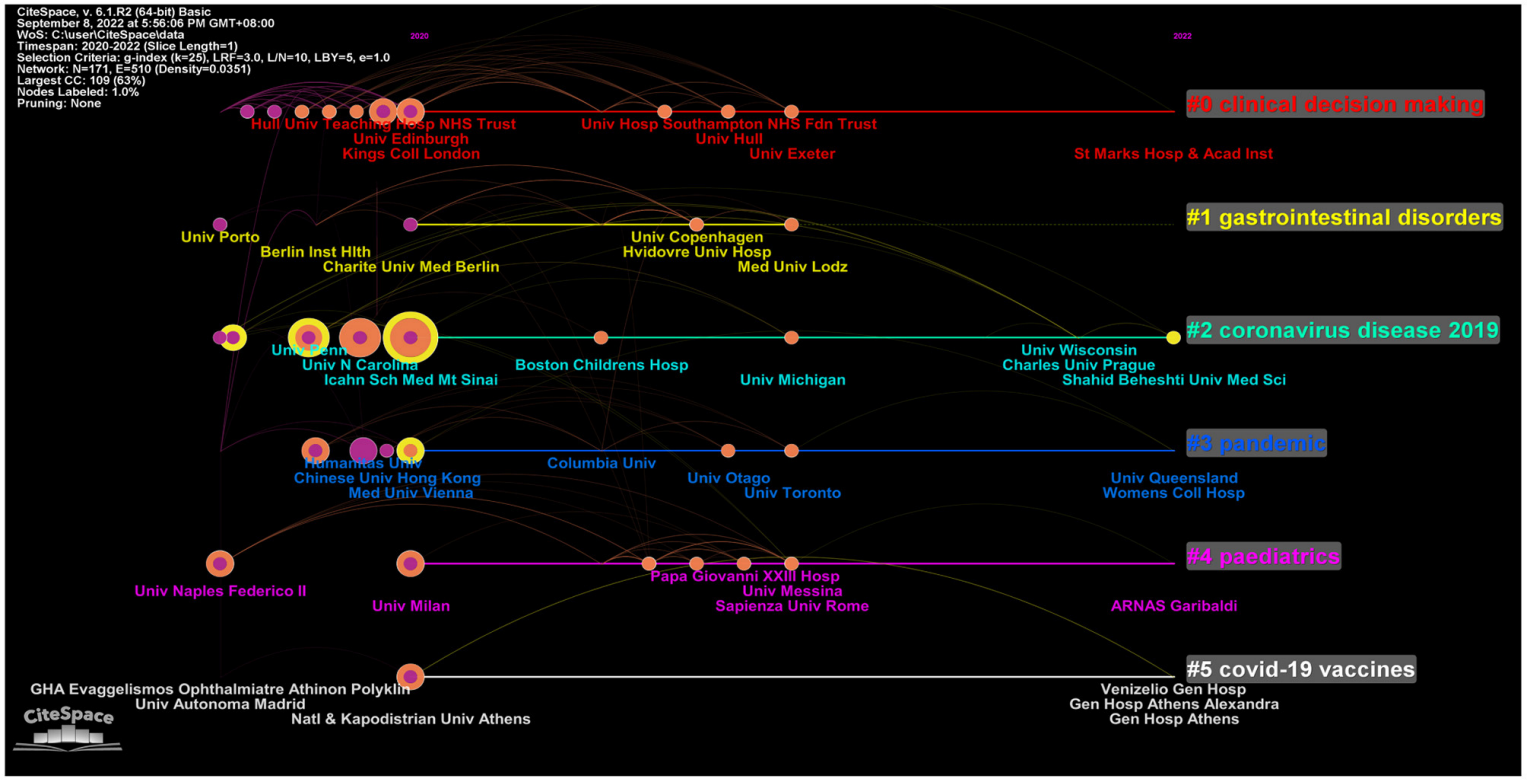
Network visualization of cooperative institutions in the field of IBD and COVID-19.

Figure 2 shows the collaboration between the institutions involved in the study. A total of 1,056 institutions were involved, and 19 met the threshold (minimum number of documents by an institution: 7). The Icahn School of Medicine at Mount Sinai had the most connections. The University of North Carolina and the Medical University of Vienna were in second and third places, respectively. Most of the collaborating institutions were research institutions from the United States or Europe.

**Figure 3:**
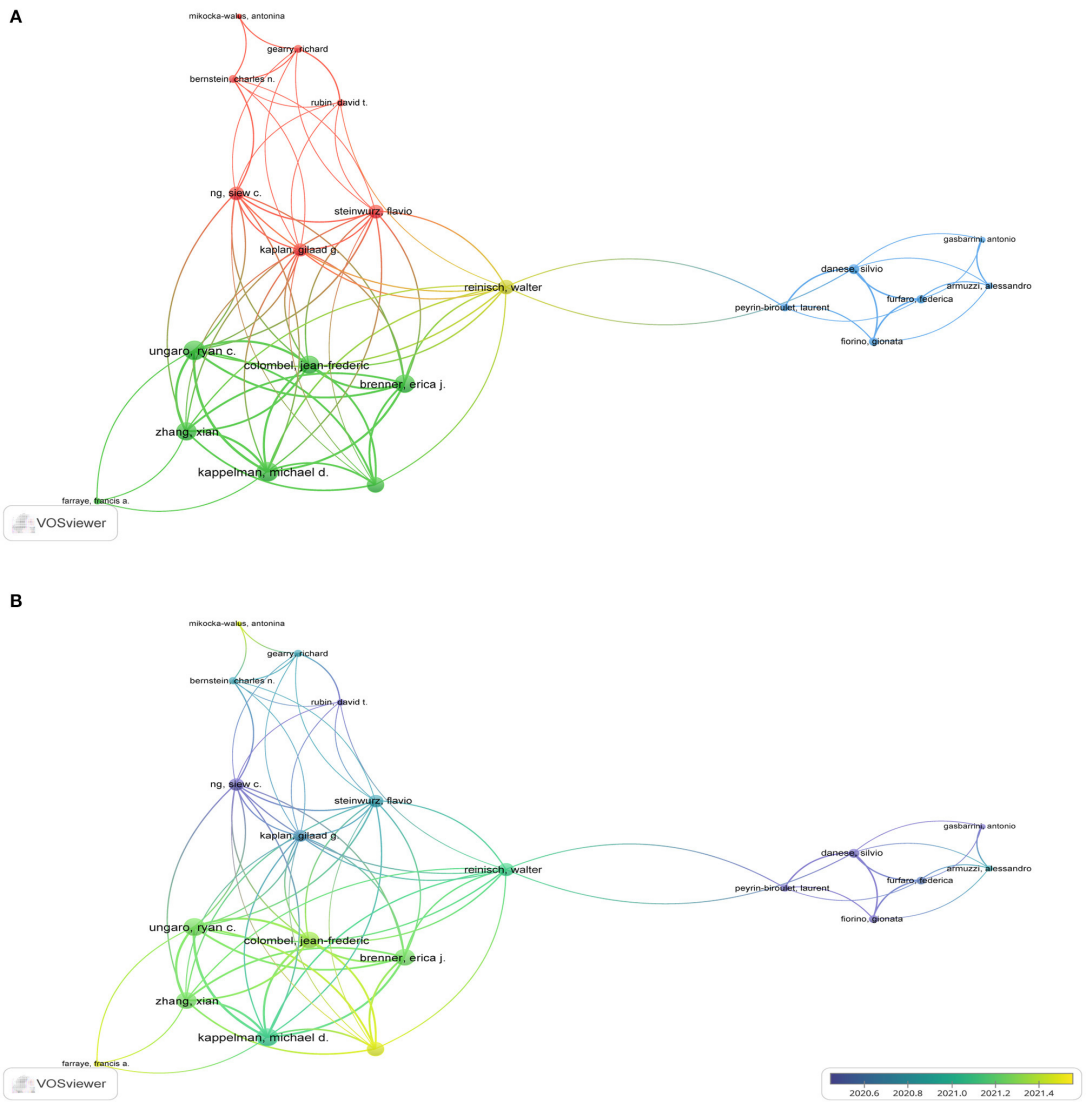
The analysis of co-authors in the field of IBD and COVID-19. **(A)** The top 21 most co-authors. **(B)** Time sequence chart of co-authors.

Figure 3 shows 21 authors in the network. The minimum number of documents per author was 5. Furthermore, Ungaro RC, Colombel JF, Brenner EJ, Zhang X, Kappelman MD, and Agrawal M collaborated the most. These authors produced several co-authored publications. Some example publications were “Data visualization in the era of COVID-19: An interactive map of the SECURE-IBD registry”, in 2020 in the *American Journal of Gastroenterol* and “Effect of IBD medications on COVID-19 outcomes: results from an international registry”, published in 2021 in *Gut*. They conducted comprehensive research on the registry, management, incidence, treatment strategies, and immunization vaccines for IBD patients in the COVID-19 pandemic context.

### 3.5. Analysis of highly co-cited references

**Figure 4:**
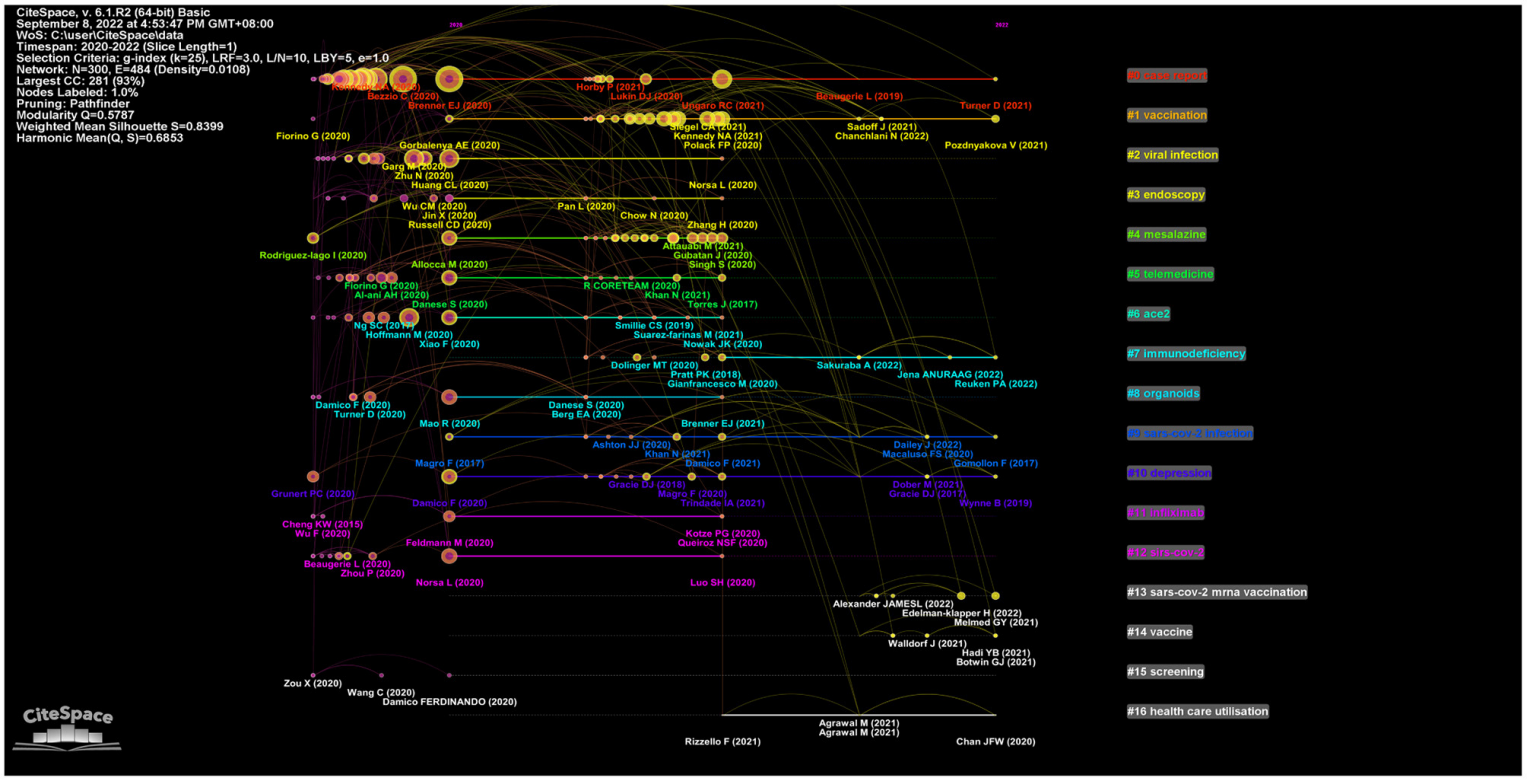
The cluster analysis of highly co-cited references in the field of IBD and COVID-19.

The term co-cited refers to references that are cited together in another publication. In this study, CiteSpace was used to detect co-cited references. Figure 4 presents a network diagram of highly co-cited references, with the time slice set to 1 year. Timeline views of co-citation references can reflect the temporal characteristics of research hotspots in the research areas concerned. By using latent semantic indexing (LSI), nominal terms were extracted from the keywords to name these clusters. Among the 17 clusters extracted, the #0 case report of COVID-19 pneumonia associated with IBD was the earliest conducted research. The top-ranked article by the number of citations was that by Brenner et al. (31). The author aimed to characterize the clinical course of COVID-19 among IBD patients and evaluate the association among demographics, clinical characteristics, and immunosuppressant treatments on COVID-19 outcomes.

### 3.6. Keywords co-occurrence and clusters analysis

**Figure 5:**
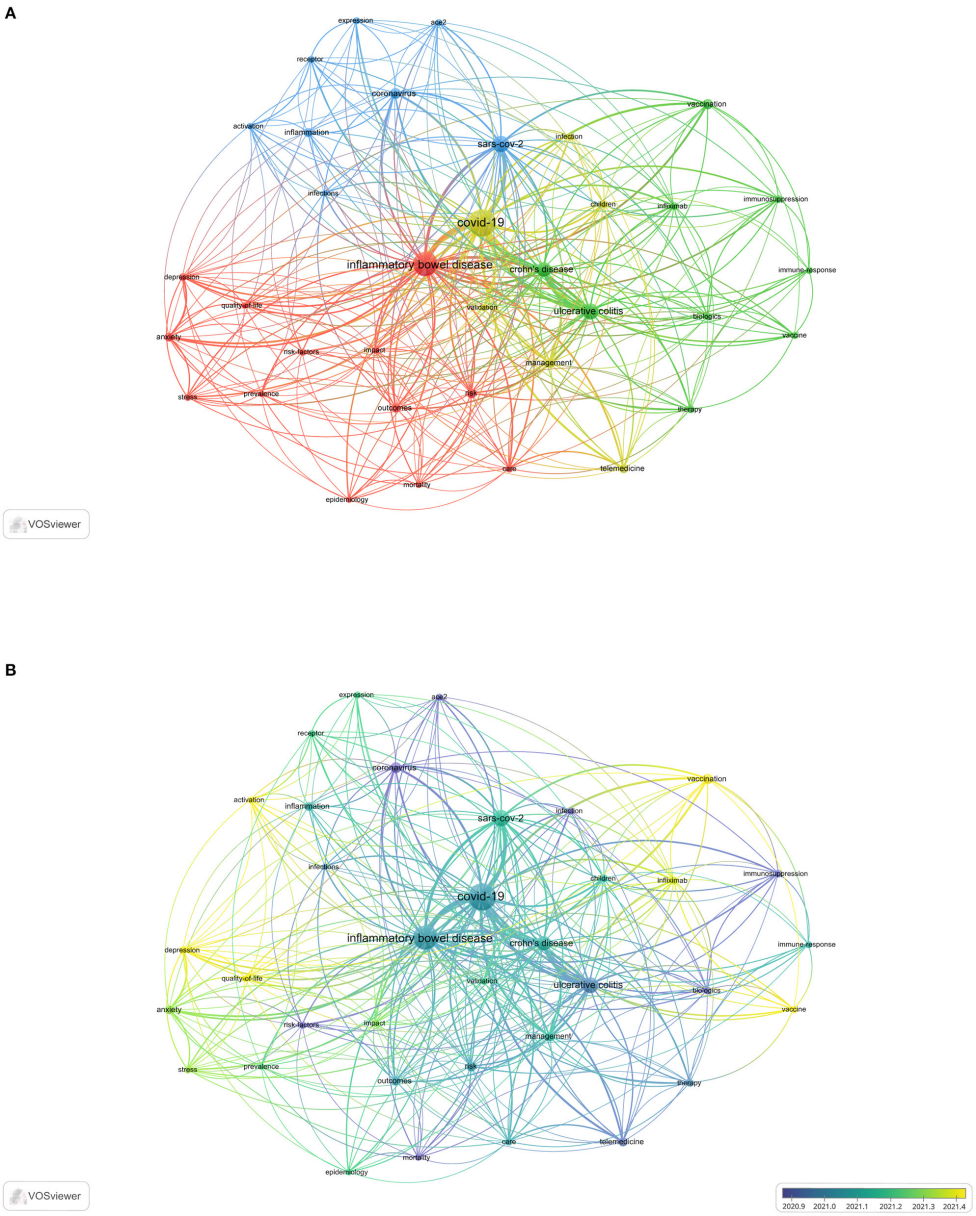
The co-occurring keywords in the field of IBD and COVID-19. **(A)** High-frequency keywords. **(B)** Time sequence chart of keyword clusters.

Keywords represent the core of an article and the essence of the article's summary. High-frequency keywords are often used to determine a hot topic in a research field. In this study, 1,362 keywords were considered. A total of 36 keywords with a frequency of 10 and above were extracted and analyzed using VOSviewer. The four most common keywords were “management”, “impact”, “vaccination”, and “receptor” (Figure 5A). Four clusters were obtained from the analysis. Cluster 1 (shown in yellow) comprised 6 items that represented the relationship between management and IBD patients; it included keywords such as “management”, “children”, and “telemedicine”. Cluster 2 (shown in red) comprised 13 items that represented the impact of COVID-19 on IBD patients; it included keywords such as “impact”, “outcomes”, “anxiety”, “depression”, “risk”, “care”, “stress”, and “quality of life”. Cluster 3 (shown in green) comprised 9 items that represented the relationship between vaccination and IBD patients; it included keywords such as “vaccination”, “biologics”, “immune response”, “immunosuppression”, and “infliximab”. This cluster (shown in blue) comprised 6 items related to the relationship between the receptor and IBD; it included keywords such as “angiotensin converting enzyme 2 (ACE2)”, “activation”, “inflammation”, and “expression”.

**Figure 6:**
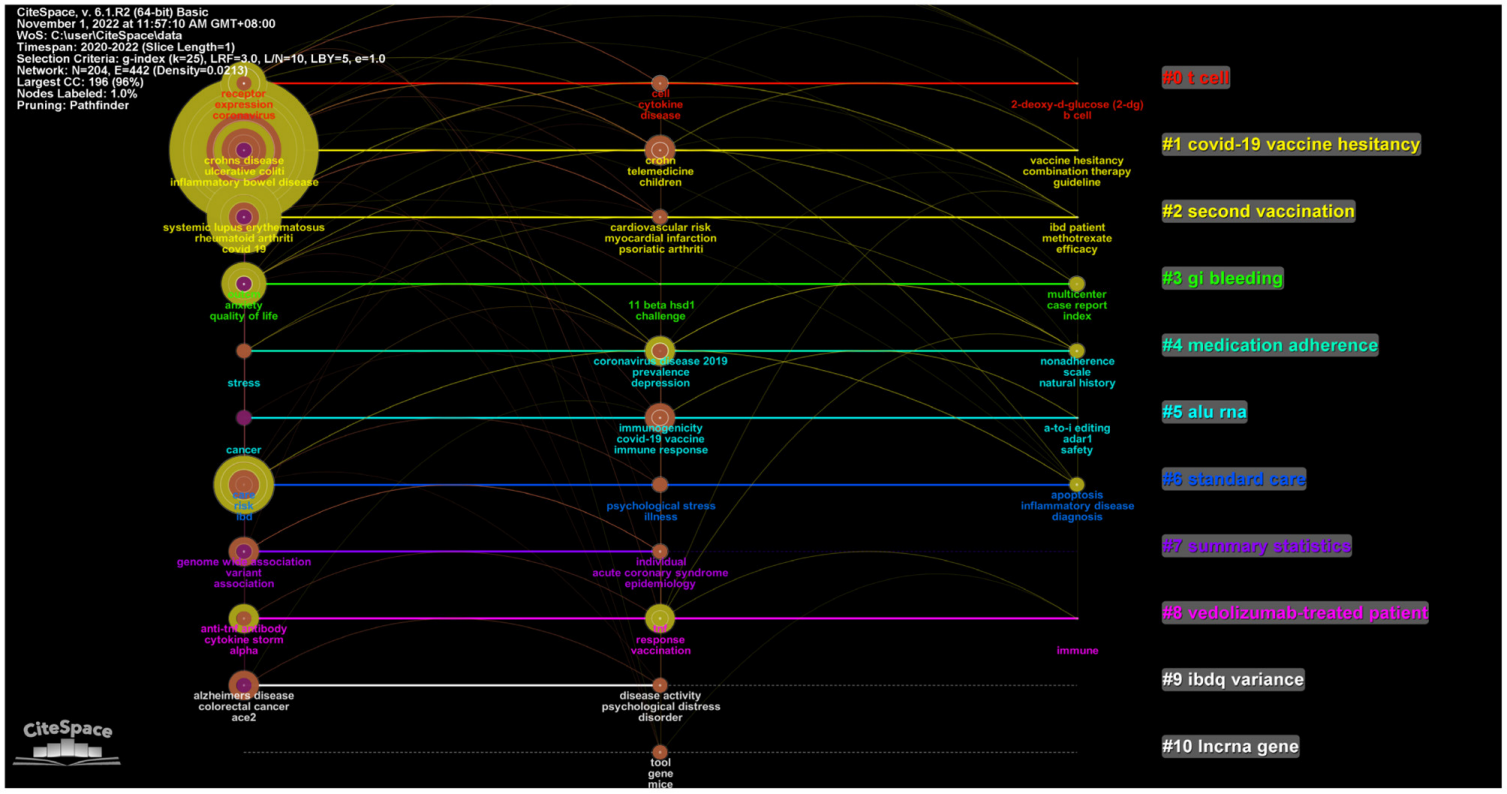
The cluster analysis of keywords in the field of IBD and COVID-19.

CiteSpace was also used to conduct keyword cluster analysis to strengthen the reliability of the keywords. Keyword nodes contained in “COVID-19 vaccine hesitancy” (Cluster #1) and “second vaccination” (Cluster #2) appeared late on the horizontal axis of time, demonstrating that these two clusters came to be novel research hotspots much later (Figure 6).

Figure 5B illustrates the evolution of keywords over time. Keywords in blue indicate that their average year of publication was earlier. In contrast, keywords in yellow indicate new research hotspots. “ACE2”, “risk-factors”, “mortality”, “infection”, “immunosuppression”, “biologics”, “therapy”, and “telemedicine” were the most commonly used keywords in 2020. Furthermore, “vaccination”, “infliximab”, “activation”, “depression”, and “quality-of-life” have been the most commonly used keywords since April 2021.

### 3.7. Analysis of the literature on the management of COVID-19 in IBD patients

Owing to the COVID-19 pandemic, the management of IBD patients has undergone an unprecedented shift. Globally, more IBD patients were infected with COVID-19, making it increasingly important to know how IBD medications could be managed during convalescence. Therefore, articles related to the management of IBD patients were screened for analysis.

**Figure 7:**
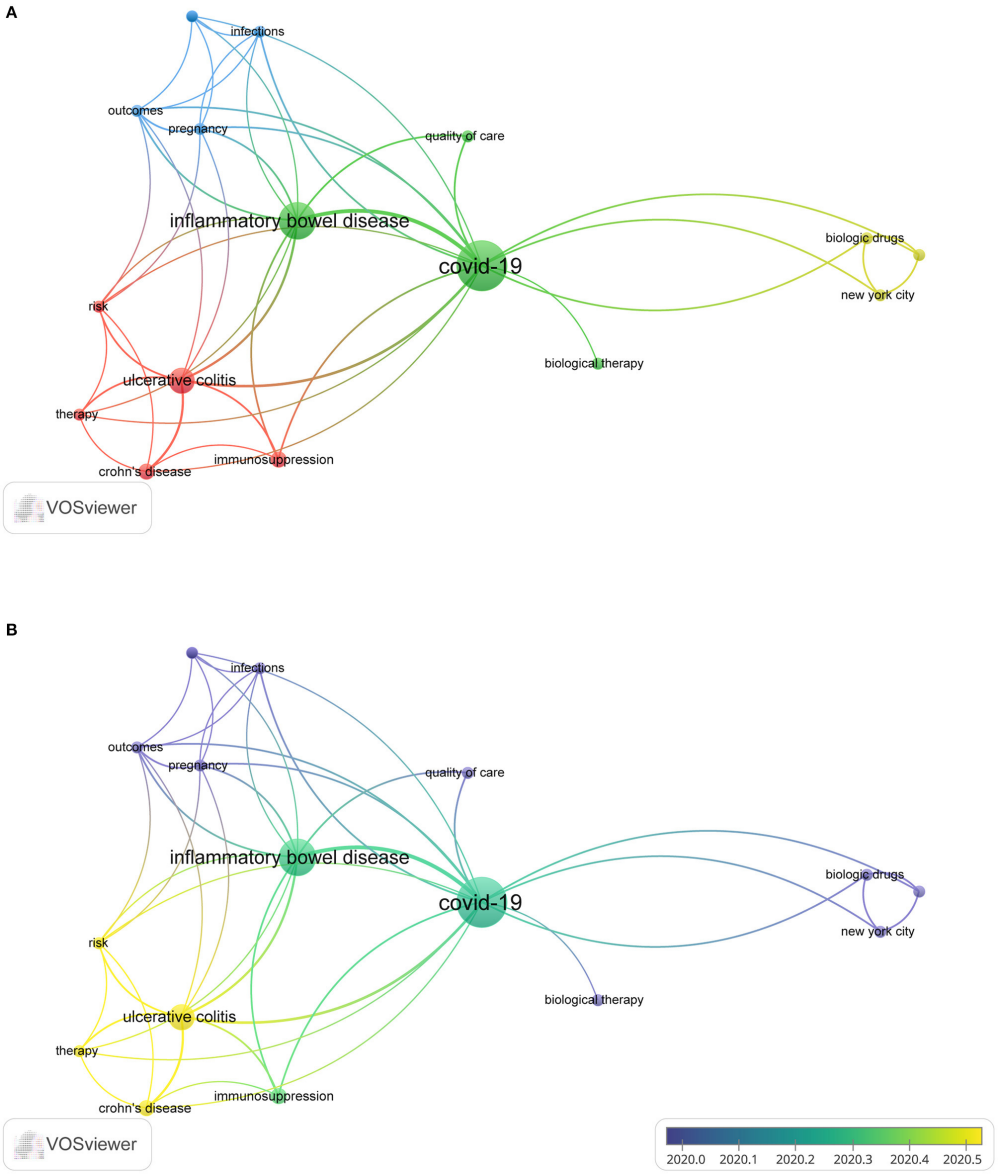
The co-occurrence of keywords **(A)** and time sequence chart of keywords **(B)** of the literature on the management of COVID-19 on IBD patients.

Supplementary Table S2 lists the top 10 highly cited papers. The most cited article was “British Society of Gastroenterology guidance for management of inflammatory bowel disease during the COVID-19 pandemic (32)” by Kennedy et al. (32) in *Gut*. During the COVID-19 outbreak, the British Society of Gastroenterology (BSG) quickly mobilized IBD centers to keep IBD patients safe. They used the best available data and expert opinion to generate a risk grid, classifying IBD patients into the highest, moderate, and lowest risk categories. The article “AGA Clinical Practice Update on Management of Inflammatory Bowel Disease During the COVID-19 Pandemic: Expert Commentary (33)” by Rubin et al. (33) had 127 citations. Both publications provided optimal guidance for managing IBD patients at the time. A total of 18 keywords were considered in the analysis (occurrence > 2) (Figure 7). The five most common keywords about IBD and COVID-19 management were “immunosuppression”, “pandemic”, “outcomes”, “pregnancy”, and “risk”.

### 3.8. Analysis of the literature on the impact of COVID-19 in IBD patients

The keyword analysis indicated that the impact of COVID-19 on IBD patients was a hot topic. Therefore, articles related to the impact of COVID-19 on IBD patients were also reviewed.

**Figure 8:**
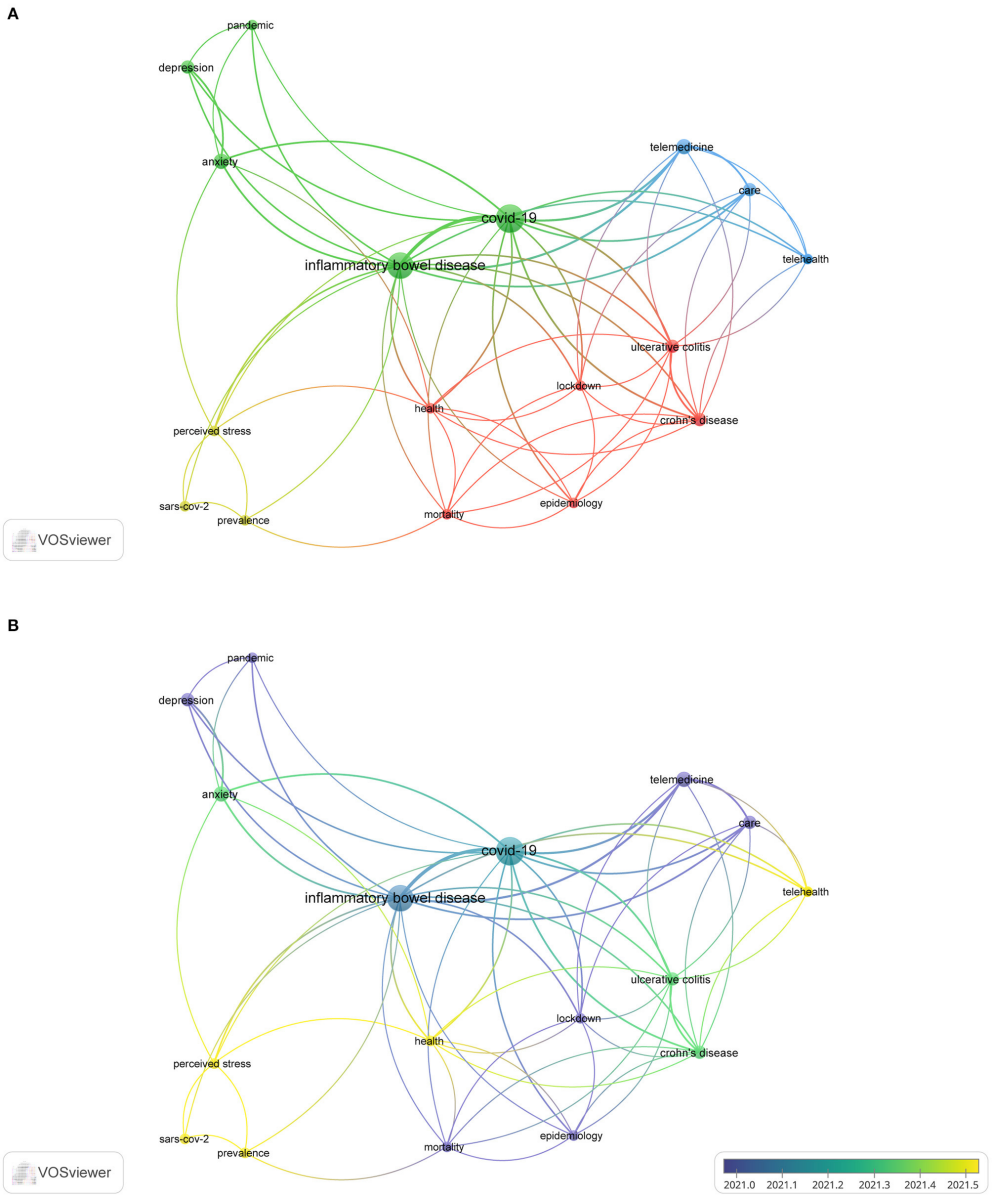
The co-occurrence of keywords **(A)** and time sequence chart of keywords **(B)** of the literature on the impact of COVID-19 on IBD patients.

Supplementary Table S3 lists the top 10 highly cited papers. “Impact of COVID-19 pandemic on the daily management of biotechnological therapy in inflammatory bowel disease patients: Reorganizational response in a high-volume Italian inflammatory bowel disease center (34)” published in the *United European Gastroenterology Journal* had 32 citations. This study was conducted in a high-volume IBD center and presented the first detailed observational report about the short-term impact of COVID-19 on patient organization and management. A total of 17 keywords were analyzed (occurrence > 2) (Figure 8). Four keywords had frequencies > 3, including the terms “telemedicine”, “anxiety”, “care”, and “depression”.

## 4. Discussion

Global medical research has focused on COVID-19 since the initial weeks of the pandemic. New population-based data suggest that IBD is a risk factor for unfavorable COVID-19 outcomes compared with the background population (35). A search of the WoSCC database revealed that 396 studies were published from 2020 to 2022. To date, the pathogenesis of IBD and COVID-19 has not been thoroughly studied. Significant scope remains for exploration and development in these areas of research.

The most co-cited references represent the most influential articles in their respective areas (36–41). The references shown in Figure 4 provided important information about IBD during the COVID-19 period. Cluster #0 focused on IBD and COVID-19 outcomes and findings. Brenner EJ's work on the outcome of COVID-19 had the highest number of citations, indicating that other researchers widely accepted this work. Brenner EJ published “Corticosteroids, but not TNF Antagonists, are Associated with Adverse COVID-19 Outcomes in Patients with Inflammatory Bowel Diseases: Results from an International Registry (31)” in 2020 in *Gastroenterology*. This article highlighted several important findings about IBD and COVID-19. TNF antagonists do not appear to be associated with severe COVID-19 (31). It was also found that increasing age, comorbidities, and corticosteroids are associated with severe COVID-19 outcomes among patients with IBD (31). “Effect of IBD medications on COVID-19 outcomes: results from an international registry [42” was published by Ungaro RC in 2021 in *Gut*. This article has several clinical implications. Combination therapy and thiopurines might be associated with an increased risk of severe COVID-19 (42). This finding supports the discontinuation of thiopurines in COVID-19-infected patients with IBD. Many other studies have also supported this viewpoint. Immunosuppressive medications may help lower the risk of adverse COVID-19 outcomes by minimizing the cytokine storm associated with severe COVID-19 (43, 44). Further studies are needed to evaluate the safety of mesalazine/sulfasalazine for this condition.

The field's research direction and scope can be intuitively identified by a network clustering analysis of keywords. Figures 5A, 6 show that the themes were diverse, and there were no discernible patterns, perhaps reflecting the complex relationship between IBD and COVID-19. In the initial phase of the COVID-19 pandemic, topics such as infection, outcomes, risk factors, mortality, ACE2, biologics, and immunosuppression received considerable focus. Clinicians and patients need to know if IBD patients are at a higher risk for COVID-19 infection (45). Concerns have been raised that the risk of COVID-19 infection may be increased in patients with IBD due to the possibly increased ACE2 levels that mediate the entry of SARS-CoV-2 into human cells (46). Thus far, no study has demonstrated that SARS-CoV-2 infections are more common among IBD patients (47), although some medications, in particular corticosteroids, have been associated with a greater risk of severe COVID-19 outcomes (31, 45). Subsequently, for children with IBD, telemedicine, care, stress, anxiety, management, and impact have received increasing attention. Compared to adult-onset IBD, childhood-onset IBD is more aggressive and progresses rapidly (48, 49). The COVID-19 pandemic has significantly affected the assessment and management of children with IBD (50–52). It is unclear whether children with IBD are more likely to contract SARS-CoV-2 (53, 54). Children with IBD need more attention and special care.

At this stage, researchers gradually began to look at “depression”, “quality of life in IBD patients”, “infliximab”, “COVID-19 vaccine”, and “second vaccination”. These keywords represent the hotspots of research. As a result of the global outbreak of COVID-19, countries worldwide have accelerated the development of vaccines against SARS-CoV-2. As shown in Figures 5B, 6, researchers have focused on the COVID-19 vaccine since 2021. Patients with IBD are highly advised to receive the COVID-19 vaccine (55, 56); however, the induced immune response may be reduced under some immunosuppressive therapies (57, 58). The efficacy of vaccines for people with IBD remains questionable (59). In several studies, researchers investigated whether patients with IBD receiving infliximab have an attenuated immune response to SARS-CoV-2 infection. In patients with IBD, the use of the anti-TNF agent infliximab was associated with a diminished serologic response to SARS-CoV-2 infection (60). Delayed second administration should be avoided in patients treated with infliximab, and further observational data are needed to clarify the effects of other biologics on SARS-CoV-2 vaccine immunogenicity (61). However, emergent hybrid variants have hindered vaccine neutralization capacity (62). There is a possibility that viral variants may evolve with harmful susceptibility to the immunity established by COVID-19 vaccination (63). In particular, the humoral immune response after the third vaccine dose in patients with IBD on anti-TNF therapy might not be protective against SARS-CoV-2 variants, particularly against Omicron (64, 65). The effectiveness of the third dose against SARS-CoV-2 variants in patients with IBD remains unclear. Therefore, aside from booster vaccine shots, advances in modifying and updating existing vaccines, new generation vaccines and more effective drugs and antibody-based therapies for treating COVID-19 patients are currently needed (66).

Figure 7 indicates that the terms “immunosuppression”, “pandemic”, “outcomes”, “pregnancy”, and “risk” were the hot topics of the management of IBD patients. Pregnant women with IBD are a particularly vulnerable group. Management of pregnant women with IBD during the COVID-19 pandemic is challenging (67). The SARS-CoV-2 pandemic has abruptly impacted the management of patients with IBD (68). Gastroenterologists have faced an unprecedented challenge, requiring not only adaptation of the therapeutic management of IBD patients and redefining medical priorities but also reorganization of health care facilities (68). The strategy to manage IBD patients should be individualized based on the risk associated with infection and IBD activities. Management based on general guidance and consensus statements from the British Society of Gastroenterology (32) and the International Organization for the Study of Inflammatory Bowel Disease (IOIBD) (69) can be divided into three categories: IBD patients not infected with COVID-19, IBD patients infected with COVID-19 but not showing symptoms, and COVID-19-infected IBD patients with or without bowel inflammation (33). IBD patients with a stable disease course and without SARS-CoV-2 infection should maintain ongoing immunomodulatory treatment since the risk of disease reactivation outweighs the risk of SARS-CoV-2 infection (32, 33, 69–72). Patients with stable IBD and positivity for SARS-CoV-2 infection should be considered individually, simultaneously balancing the risk of inflammatory disease reactivation with the risk of severe COVID-19 course (68). The third scenario presents the greatest challenge, as it affects both IBD and COVID-19 treatment. Depending on the severity of COVID-19 and IBD, clinicians may need to follow IBD management guidelines closely.

According to the keywords with the strongest citation bursts in Figure 8, the impact of COVID-19 on IBD patients mainly includes “telemedicine”, “anxiety”, “care”, and “depression”. The social and psychological impact of the COVID-19 pandemic is believed to be severe and long-lasting (73). Over time, fear, anxiety, and depression have grown among people (74). This condition, defined as long COVID, is now recognized as a public health priority (75). During the COVID-19 pandemic, the treatment routines of patients with chronic diseases changed significantly (76, 77). Compared to the general population, IBD patients are more likely to suffer from anxiety and depression (78). The latent psychopathological conditions will be exacerbated by self-isolation, future uncertainty, and lack of regular face-to-face contact with referred physicians (68). In the early phase of the pandemic, according to an online survey, only 11% of patients found relief from their worries from medical consultations, indicating a communication gap between patients and physicians (79). In this context, telemedicine, and perhaps telepsychology, appears pivotal. Therefore, clinical practice has been rapidly adapted to compensate for the impact of COVID-19. To assess the long-term impact of COVID-19 on IBD patients, the outcomes of patients should be carefully analyzed. To date, there are limited data regarding IBD and COVID-19, but the quality of evidence has gradually improved over the past few years. Large-scale studies on patients with IBD may provide more precise answers about the long-term impact of COVID-19 on IBD.

The analysis software VOSviewer and CiteSpace have some limitations in this work. They are limited to analyzing data downloaded from databases outside the WoSCC and are mainly suitable for analyzing English-language literature. There is some bias in the literature selection. Cluster analysis only analyzes the literature from key nodes in citation networks and cannot completely reverse the research situation of research hotspots represented by clustering. To fully understand the research situation of this cluster, a large number of studies from non-key nodes need to be further analyzed. These factors may lead to some differences between the research results and the actual situation. Therefore, we still need to pay attention to the latest research results and literature from other databases in future research to better grasp the development direction and trend of this field.

The bibliometric analysis reveals that research on IBD and COVID-19 is developing rapidly at present. Cluster analysis of keywords found that several research topics have been formed in this field, such as “management”, “impact”, “vaccination”, and “receptor”. The “depression”, “quality of life”, “infliximab”, “vaccination”, and “second vaccination” in the field of IBD and COVID-19 have received much attention recently. Future research should further explore the immune response to COVID-19 vaccination in biologically patients, the psychological impact of COVID-19, IBD management guidelines, and the long-term impact to COVID-19 in IBD patients. More clinical studies and basic research should be conducted in the future to clarify the relationship between IBD and COVID-19. This will provide theoretical and practical guidance on how to intervene when IBD patients receiving immunotherapy are infected with COVID-19.

## Data availability statement

The original contributions presented in the study are included in the article/Supplementary material, further inquiries can be directed to the corresponding author.

## Author contributions

FW performed the data curation and wrote the original draft of the manuscript. HX and JX performed the statistical analysis. YX performed the supervision, reviewed, and edited the manuscript. All authors contributed to the article and approved the submitted version.
