# Supplementary material for: A bibliometric analysis of inflammatory bowel disease and COVID-19 researches
Source: Front Public Health. 2023 Jan 30;11:1039782. doi: 10.3389/fpubh.2023.1039782 (PMC9922853; doi:10.3389/fpubh.2023.1039782)
Supplement: Supplementary file 1 [file Table_1.docx]

**Table S1.** The 10 most cited papers in IBD and COVID-19.

| Rank | Title | Journal | Year | TLCS/TGCS |
| --- | --- | --- | --- | --- |
| 1 | Corticosteroids, But Not TNF Antagonists, Are Associated with Adverse COVID-19 Outcomes in Patients with Inflammatory Bowel Diseases: Results from an International Registry | Gastroenterology | 2020 | 91/388 |
| 2 | British Society of Gastroenterology guidance for management of inflammatory bowel disease during the COVID-19 pandemic | Gut | 2020 | 45/159 |
| 3 | COVID-19 and immunomodulation in IBD | Gut | 2020 | 30/152 |
| 4 | Effect of IBD medications on COVID-19 outcomes: results from an international registry | Gut | 2021 | 37/140 |
| 5 | AGA Clinical Practice Update on Management of Inflammatory Bowel Disease During the COVID-19 Pandemic: Expert Commentary | Gastroenterology | 2020 | 36/127 |
| 6 | Are Patients with Inflammatory Bowel Disease at Increased Risk for Covid-19 Infection? | Journal of Crohns & Colitis | 2020 | 34/115 |
| 7 | Infliximab is associated with attenuated immunogenicity to BNT162b2 and ChAdOx1 nCoV-19 SARS-CoV-2 vaccines in patients with IBD | Gut | 2021 | 29/95 |
| 8 | Expression of SARS-CoV-2 Entry Molecules ACE2 and TMPRSS2 in the Gut of Patients With IBD | Inflammatory Bowel Diseases | 2020 | 15/94 |
| 9 | Pediatric Crohn Disease and Multisystem Inflammatory Syndrome in Children (MIS-C) and COVID-19 Treated with Infliximab | Journal of Pediatric Gastroenterology and Nutrition | 2021 | 8/76 |
| 10 | Intestinal Inflammation Modulates the Expression of ACE2 and TMPRSS2 and Potentially Overlaps with the Pathogenesis of SARS-CoV-2-related Disease | Gastroenterology | 2021 | 0/48 |

TLCS, Total local citation score, which is the number of times cited by other papers in the local collection; TGCS, Total global citation score, which is the citation frequency based on the full WoSCC count at the time the data was downloaded.
