# Supplementary material for: A bibliometric analysis of inflammatory bowel disease and COVID-19 researches
Source: Front Public Health. 2023 Jan 30;11:1039782. doi: 10.3389/fpubh.2023.1039782 (PMC9922853; doi:10.3389/fpubh.2023.1039782)
Supplement: Supplementary file 2 [file Table_2.docx]

**Table S2.** The papers that detail the IBD and COVID-19 management

| Rank | Title | Journal | Year | TLCS/TGCS |
| --- | --- | --- | --- | --- |
| 1 | British Society of Gastroenterology guidance for management of inflammatory bowel disease during the COVID-19 pandemic | *Gut* | 2020 | 45/159 |
| 2 | AGA Clinical Practice Update on Management of Inflammatory Bowel Disease During the COVID-19 Pandemic: Expert Commentary | *Gastroenterology* | 2020 | 36/127 |
| 3 | Impact of COVID-19 pandemic on the daily management of biotechnological therapy in inflammatory bowel disease patients: Reorganisational response in a high-volume Italian inflammatory bowel disease centre | *United European Gastroenterology Journal* | 2020 | 8/27 |
| 4 | Impact of COVID-19 on diagnosis and management of paediatric inflammatory bowel disease during lockdown: a UK nationwide study | *Archives of Disease in Childhood* | 2020 | 5/20 |
| 5 | Management of COVID-19 Pandemic in Spanish Inflammatory Bowel Disease Units: Results from a National Survey | *Inflammatory Bowel Diseases* | 2020 | 5/11 |
| 6 | Practical management of inflammatory bowel disease patients during the COVID-19 pandemic: expert commentary from the Gastroenterological Society of Australia Inflammatory Bowel Disease faculty | *Internal Medicine Journal* | 2020 | 1/10 |
| 7 | Questionnaire assessment helps the self-management of patients with inflammatory bowel disease during the outbreak of coronavirus Disease 2019 | *Aging-US* | 2020 | 5/10 |
| 8 | Activities related to inflammatory bowel disease management during and after the coronavirus disease 2019 lockdown in Italy: How to maintain standards of care | *United European Gastroenterology Journal* | 2020 | 0/9 |
| 9 | Impact of COVID-19 pandemic on the management of paediatric inflammatory bowel disease: An Italian multicentre study on behalf of the SIGENP IBD Group | *Digestive and Liver Disease* | 2021 | 4/8 |
| 10 | Worldwide Management of Inflammatory Bowel Disease During the COVID-19 Pandemic: An International Survey | *Inflammatory Bowel Diseases* | 2021 | 0/4 |

TLCS, Total local citation score, which is the number of times cited by other papers in the local collection; TGCS, Total global citation score, which is the citation frequency based on the full WoSCC count at the time the data was downloaded.
