# Supplementary material for: A bibliometric analysis of inflammatory bowel disease and COVID-19 researches
Source: Front Public Health. 2023 Jan 30;11:1039782. doi: 10.3389/fpubh.2023.1039782 (PMC9922853; doi:10.3389/fpubh.2023.1039782)
Supplement: Supplementary file 3 [file Table_3.docx]

**Table S3.** The papers that detail the impact of COVID-19 on IBD patients

| Rank | Title | Journal | Year | TLCS/TGCS |
| --- | --- | --- | --- | --- |
| 1 | Impact of COVID-19 pandemic on the daily management of biotechnological therapy in inflammatory bowel disease patients: Reorganisational response in a high-volume Italian inflammatory bowel disease centre | *United European Gastroenterology Journal* | 2020 | 8/27 |
| 2 | Impact of COVID-19 on diagnosis and management of paediatric inflammatory bowel disease during lockdown: a UK nationwide study | *Archives of Disease in Childhood* | 2020 | 5/20 |
| 3 | Impact of the COVID-19 outbreak and the serum prevalence of SARS-CoV-2 antibodies in patients with inflammatory bowel disease treated with biologic drugs | *Digestive and Liver Disease* | 2021 | 4/15 |
| 4 | A cross-sectional survey on the psychological impact of the COVID-19 pandemic on inflammatory bowel disease patients in Saudi Arabia | *Saudi Journal of Gastroenterology* | 2020 | 3/14 |
| 5 | Epidemiology and the Impact of Therapies on the Outcome of COVID-19 in Patients with Inflammatory Bowel Disease | *American Journal of Gastroenterology* | 2020 | 5/13 |
| 6 | Incidence, outcomes, and impact of COVID-19 on inflammatory bowel disease: Propensity matched research network analysis | *Alimentary Pharmacology & Therapeutics* | 2022 | 1/8 |
| 7 | Impact of COVID-19 pandemic on the management of paediatric inflammatory bowel disease: An Italian multicentre study on behalf of the SIGENP IBD Group | *Digestive and Liver Disease* | 2021 | 4/8 |
| 8 | Impact of COVID-19 outbreak on the care of patients with inflammatory bowel disease: A comparison before and after the outbreak in South China | *Journal of Gastroenterology and Hepatology* | 2021 | 0/7 |
| 9 | Clinical Outcomes of COVID-19 and Impact on Disease Course in Patients with Inflammatory Bowel Disease | *Canadian Journal of Gastroenterology and Hepatology* | 2021 | 0/3 |
| 10 | Impact of the COVID-19 pandemic on inflammatory bowel disease: The role of emotional stress and social isolation | *Stress and Health* | 2022 | 3/3 |

TLCS, Total local citation score, which is the number of times cited by other papers in the local collection; TGCS, Total global citation score, which is the citation frequency based on the full WoSCC count at the time the data was downloaded.
